# Editorial: Managing and mitigating suffering at work

**DOI:** 10.3389/fpsyg.2022.1033440

**Published:** 2022-10-24

**Authors:** M. Isabel Sánchez-Hernández, Živilė Stankevičiūté, Rūta Adamonienė, Martina Blašková

**Affiliations:** ^1^Department of Business Administration and Sociology, School of Economics and Business Administration, University of Extremadura, Badajoz, Spain; ^2^School of Economics and Business, Kaunas University of Technology, Kaunas, Lithuania; ^3^Mykolas Romeris University, Public Security Academy, Kaunas, Lithuania; ^4^Department of Management and Informatics, Faculty of Security Management, Police Academy of the Czech Republic in Prague, Prague, Czechia

**Keywords:** burnout, job insecurity, techno-stress, suffering, wellbeing, workplace

## Introduction

This special issue of the Journal Frontiers in Psychology, in the section on Organizational Psychology, is devoted to the multi-faceted emergent concept in the current world of work of employees' suffering. We define suffering at work as a destabilizing psychological experience arising when employees run into insuperable and tenacious barriers, after having used up all their available resources trying to improve a situation, looking for wellbeing in the workplace, or a better organization of work with regards to quality and safety.

Unfortunately, different factors could negatively affect the working experience in highly volatile, uncertain, complex, and ambiguous (VUCA) contexts. For instance, and even considering that the implementation of teleworking was a security practice to face the crisis resulting from the COVID-19 pandemic, the increasing use of technology has led to the worsening of technostress among workers. In the same line, the current organizational downsizing processes are also causing suffering, as has been evidenced in managers' mental health, employees' job insecurity, or affective disorders of employees such as depression or anxiety.

Researchers are becoming increasingly interested in understanding suffering at work, and practitioners are conscious about the organizational problems associated with it. Employees suffering at work can lead to lower productivity, negative deviant behaviors, lost workdays, and a higher staff turnover. Managers, supervisors, or employers could help lower workplace suffering. New trends in Human Resources Management (HRM) contribute to combatting suffering such as responsible job designs or mindfulness, but there is still a lack of evidence, both theoretical and empirical, regarding how to manage suffering. To fill the gap, the twenty-one articles published in this special issue present the recent advances on the topic and make a significant contribution to a better understanding of managing and mitigating suffering at work.

As a whole, the published manuscripts address three specific areas of interest, namely sources of suffering, consequences, and potential solutions, that are presented and commented as follows.

## Sources of suffering

Although a huge variety of job demands might serve as sources of suffering at work, this special issue focuses on seven antecedents, namely job insecurity, abusive supervision, stress and technostress, workplace violence, abusive and inefficient red tape, unsatisfactory personal relations, and role-overload.

### Job insecurity

Job insecurity, which refers to a perceived threat to the continuity and stability of employment as it is currently experienced, is considered as a hindrance stressor and one of the huge issues in today's contemporary working life. Job insecurity impedes employees' overall functioning, personal growth, and development, and is more problematic for their wellbeing than the certainty of dismissal. Moreover, job insecurity causes negative consequences not only for employees, but also for businesses, leading to lower organizational performance.

Drawing upon the strength model of self-control, He et al. examined whether job insecurity was harmful to different types of proactive behaviors of employees. Based on two-wave data collected from 227 employees in China, the authors concluded that job insecurity was negatively associated with two types of proactive behaviors, namely individual-oriented and organization-oriented proactive behaviors. While acknowledging that complete elimination of job insecurity is infeasible for organizations, the paper reveals that the role of future work self-salience and socioeconomic status, as motivational and situational factors, can independently and interactively moderate the relationship between job insecurity and proactive behavior. These findings not only extend the strength model of self-control, but also encourage the organizations to develop differentiated management strategies for different employees.

The paper by Stankevičiute et al. deals with the link between job insecurity and its potential outcomes referring to the current volatility, uncertainty, complexity, and ambiguity (VUCA) context. Firstly, treating job insecurity as a hindrance stressor, the paper claims a negative association between job insecurity and trust in the organization, subjective wellbeing, and task performance. Secondly, the paper focuses on the relationship between constructs in the virtuous cycle, analyzing how trust in the organization, subjective wellbeing, and task performance are related. The authors suggest that the complex of actions with respect to employee development, transparent communication, taking part in decision-making, and the increase in organizational justice of the business actions might create a synergic effect and reduce job insecurity as such.

### Abusive supervision

The study by Gul et al. focused on knowledge-hiding behaviors and abusive supervision. In their paper, social exchange theory was employed as the main theoretical framework predicting knowledge concealing in the presence of abusive supervision. Based on empirical results derived from a study conducted in Pakistan which received 340 responses, it was demonstrated that abusive supervision was positively related to three types of employees' knowledge-hiding behavior, namely evasive hiding, playing dumb, and rationalized hiding behaviors. Further, the paper provided evidence that psychological contract breach partially mediated the abusive supervision-knowledge hiding behavior linkage. Finally, the core contribution of the paper lies in the approach tackling the negative aspect, namely knowledge-hiding instead of the positive aspect, i.e., knowledge-sharing.

### Stress and technostress

The stress produced by the massive lay-offs to adjust the labor market to the new market situation is an object of study as a source of suffering at work, especially in sectors more susceptible to modernization and restructuration processes, like the financial one. Robina-Ramírez et al. study how a sample of 601 employees from the banking sector in Spain have faced this adversity. In adversity at work, the authors focus their attention on promoting values, more specifically employees' spirituality and transcendence, for a better adaptation of workers to the pressure and stressful tasks at the companies.

Another source of suffering at work is technostress, which can affect the individual, group, and professional sphere of the sufferer, and also be a cause of burnout. Specifically, hyper-connection to different devices because of the COVID-19 pandemic situation has left workers susceptible to exhaustion. Also, under the Conservation of Resources as a theoretical framework, a multidimensional scale of technostress with four dimensions (anxiety, fatigue, skepticism, and ineffectiveness) is verified by Buenadicha-Maetos et al. in a sample of 333 university students representing the next generations at work. This work highlights the dark side of technology if is not well managed. In addition, this work demonstrates that perceived stress and the individual students' conflicts have a mediation effect between technostress and emotional exhaustion.

Considering the perceptions of 339 Dutch childcare workers, the study by Bauwens et al. tests a model in which technostress influences the quality of care delivered, and the relationship is mediated by the emotional exhaustion of workers. In the model, empowering leadership is considered a moderator variable to be managed for stimulating employees' responsibility and accountability for the different dimensions of technostress.

### Workplace violence

Workplace violence, especially in the health sector, is a common problem around the globe. The study by Kader et al. explores factors that influence incidents of violence against healthcare professionals in Bangladesh, analyzing the content of 157 incidents reported by doctors on social media. Findings show that the analyzed primary care centers experienced more violence than other facilities, largely due to insufficient human resources to meet patients' demands and expectations.

### Red tape

New compulsory procedures and regulations are dramatically mushrooming at work, being categorized as red tape. Instead of improving job satisfaction, they lack functionality and generate suffering. In healthcare organizations, Muylaert et al., with a sample of 277 head nurses in elderly care homes in Flanders, empirically prove that red tape undermines head nurses' job satisfaction and discretionary room acts as an underlying mechanism in this process. This relationship is weaker when autonomous motivation is higher.

### Poor personal relations and opportunistic behavior

The study by Yuan and Gao determines the main causes of employees' complaints in China with two complementary samples of 268 and 349 workers respectively. The main conclusion of the research indicates that dissatisfaction with personal relations is the most significant problem.

Also in China, the work of Chen et al. investigates the influence of opportunistic behavior tolerance on response strategy selection in a sample of 206 border agents in channel transactions. The authors focus their attention on the fact that channel transactions are increasing, and there is also an increase in negative acts of channel's members seeking self-interest. Some channel members suffer at the expense of other channel members' interests to maximize benefits, including fraud, breach of contract, dishonesty, and distortion of facts.

### Role-overload

Generally, role overload is characterized by work demands or a set of responsibilities that exceeds the employees' time, energy, and capabilities. The work of Tang and Vanderberghe, which will be commented on in the next section, considers role-overload as a source of suffering at work.

## Consequences of suffering

Recently, employee turnover has become one of the most relevant concerns for organizations. This special issue offers works devoted to analyzing specific consequences of suffering at work that are connected in a negative circle: underperformance, deviant work behavior, and employee turnover.

### Underperformance

Role overload, considered as a challenge stressor, has been analyzed in the work of Tang and Vandenberghe to demonstrate that it undermines performance. To mitigate this negative effect, the authors propose that this relation must be buffered by leader-member exchange. Two complementary studies, and a supplementary panel, are presented involving a total of 502 customer-service employees in Canada. This research accentuates the scope of consequences of role overload by examining various aspects of performance such as in-role performance, job dedication, voice behavior, and reward recommendations. It is confirmed that role overload not only harms the individual, since it may engender such severe psychological strain as depression, but it can also threaten the organization, indirectly undermining work performance, particularly when leader-member exchange is low.

### Deviant work behavior

According to Memon et al., deviant work behavior is considered to be a consequence of suffering at work, referring to counterproductive work behaviors, turnover intention, and prohibitive voice behavior. As such behaviors can also have significant negative effects on organizations, the authors addressed the potential measures for decreasing them. The results obtained from 385 employees of 40 large manufacturing organizations operating in Pakistan revealed that both internal and external CSR contributed to the reduced level of deviant work behaviors. Additionally, job satisfaction fully mediated the relationship for internal CSR while partially mediating for external CSR.

### Employee turnover

Sometimes preceded by deviant work behavior, one of the consequences of suffering at work is the intention to quit the company. Turnover intention reflects an employee's inclination to search for alternative employment and is considered a direct and essential predictor of an organization's actual turnover. Employee turnover is a major concern for many organizations around the world as turnover usually negatively affects the performance and profitability of the organization, and increases the chances of losing good employees. Still, adopting appropriate management practices allows organizations to find ways to lower employee turnover. Along this line, Awan et al. argue and demonstrate through a sample of 220 bank employees in Pakistan that role conflict and job embeddedness are opposing factors that lead workers to quit or remain within the company.

Returning to work after a serious illness or injury that necessitates time off work and a subsequent re-engagement with the work environment is also a cause of suffering. The paper by Woods and Matthewson explores how the process is negotiated and executed in Australia, according to the workers' compensation legislation. It could be supportive and successful, or it could exacerbate the suffering of returning workers. The authors also discuss how the suffering that workers experience could be mitigated, describing several factors like alignment of worker, the advocacy provided by the return-to-work coordinator, and employer expectations.

### Potential solutions for mitigating suffering at work

The current publications reveal several important aspects. First, work-life balance, meaningful work, mindfulness, and organizational culture can serve for organizations as the means for reducing suffering at work. Second, by eliminating or reducing suffering at work, organizations will better control its negative effects such as employee turnover.

### Promoting work-life balance

Work-life balance refers to obtaining a sufficient degree of satisfaction at both home and work. Usually, achieving a satisfactory work-life balance is understood as restricting one side (usually work), to have more time for the other. In recent decades, a great deal of emphasis has been placed on work-life balance, as improving work–life balance is generally linked to employees' motivation, satisfaction, and engagement at the individual level, and higher productivity and organizational competitiveness at the organizational level. In contrast, the impossibility to reconcile professional and personal life is a source of suffering for employees, causing problems at the medium term to the organization as a whole.

The paper by Lonska et al. evaluates the flexibility of reconciling work and private life for Latvian employees during the COVID-19 pandemic. The authors analyze the work–life balance requirements and notice that they are highly dependent on the individual's personal circumstances. To avoid gender gaps, flexibility is desirable for both women and men.

### Creating meaningful work

Recently, more individuals are desiring meaningful work. In general, meaningful work captures three elements: significance, broader purpose, and self-realization. As meaningful work positively correlates with many important individual work and career outcomes, such as work engagement or career development, more and more organizations are engaged in fostering meaningful work by implementing diverse practices. Moreover, meaningful work has the potential to reduce the harm employees experience at work. In line with this, Tariq et al. explore envy at work as a cause of suffering and highlight that meaningful work will help employees to regulate its negative effects. The empirical work, with a sample of 439 employees in Pakistan from four famous fast-food companies, demonstrates that a narrow span of supervision will increase work engagement and reduce envy.

### Practicing mindfulness

Tu et al. defend that mindfulness practice could reduce burnout. This article, co-authored by researchers from China and the USA, has put the attention on 537 social workers in China, and examines the relations existing between job demands, resources, and burnout. This investigation has resulted in the suggestion that job demands and resources really affect burnout, especially through both health and motivation impairment. High job demands were linked to high burnout while high job resources were linked to a reduction in burnout. This emphasizes and calls for applying mindfulness practice not only among social workers but also among other work categories and groups.

It is obvious that the topic of wellbeing is increasingly penetrating the education sector too. Along this line, the study of Song et al. searched for the links between basic psychologic needs and positive emotions of 398 Chinese preschool teachers and confirmed the hypothesis that trait mindfulness can predict and improve job satisfaction. Results have accented that basic psychological needs and positive emotions play a sequential intermediary role between preschool teachers' trait mindfulness and job satisfaction. From the practical perspective, the managers should establish a strong emotional support system to create an environment conducive to releasing and eliminating emotions and encourage preschool teachers to carry out internal self-dialogue and positive psychological suggestion.

The study by Song and Park puts the attention on customer injustice, referring to the unfair treatment that employees experience during service encounters, such as verbal aggressions or disrespect. Authors pointed out that escalated negative customer behavior has bad impacts on frontline employees' wellbeing and the prosperity of organization and, based on the survey of 259 participants in South Korea, their hypotheses were confirmed—emotional stability and attentiveness moderate relations between customer injustice and customer-directed sabotage.

### Managing organizational culture

To manage and mitigate suffering, it is necessary to understand the type of organizational culture and its relationships with the environment. Assens-Serra et al. analyze the capacity of some environment variables, business strategies, and organizational competencies to predict the presence of specific cultures in a subsample of 362 Spanish managers, and a subsample of 1,317 Peruvian managers. Surprisingly, when compared to the literature, the authors found almost no relations between the environment variables and the culture types. In addition, strategy and competencies have a significant predictive capacity, especially in a situation of clan, hierarchy, and market culture. The article concludes showing the characteristics of the types of organizational culture that could be useful for a better management of suffering.

A further inter-continental study, the article by Kumpikaite-Valiuniene et al., connects authors from Lithuania and China to investigate the impact of different types of organization cultures on the adjustment of self-initiated expatriates. With participation from 125 expatriates around the world, their work and non-work-related adjustment was explored in very well-known types of culture: clan, adhocracy, market, and hierarchy. Although clan culture has expressed a positive effect on the expatriates' work and non-work-related adjustment, innovative culture was discovered to have a negative impact in this area. In line with the soul of this Special Issue in Frontiers in Psychology, culture types based on friendly and supportive relations and values fit the self-initiated expatriates' values and fostered their adjustment in the organization and the host country.

## Conclusion

As a result of work intensification, increased work demands, unstable employment conditions, violence at work, abusive supervision, and other work-related aspects, suffering at work has become one of the main issues in our contemporary working world. In the meantime, decent work—sought by Sustainable Development Goal number 8—and good health and wellbeing—sought by Sustainable Development Goal number 3—can be achieved only by minimizing, if not eliminating, suffering at work.

New perspectives for a better management of employees' suffering are needed to deal with present and future concerns. From the point of view of systematic efforts in the area of improving management decisions and mechanisms designed to eliminate or at least alleviate the work suffering of employees, it is extremely important to take into account relationship-procedural imperfections and from them resulting and often arising mental discomfort. The feeling of low acceptance by colleagues or even the feeling of social and environmental alienation, one's own personal and professional inadequacy, low resilience to ever-increasing demands, fear of failing to meet new challenges, etc., is expanding and affecting more and more individuals and working groups.

Natural psychological mechanisms try to warn their bearers (individuals and groups) with gentle signals at first. However, specialists need to be involved in addressing and coping with the origins of psychological and social disorders. Facilitation, psychological support, consultant assistance, psycho-hygiene centers, mechanisms for increasing tolerance, mutual understanding, and cohesion are particularly appropriate in such situations.

It should be emphasized that it is always demanding and sensitive to prepare really meaningful and systemic measures in real organizations of various industries and sizes, with a chance to help and strengthen employees mentally. This effort transcends the boundaries of a single scientific discipline, and uncompromisingly calls for an active partnership of organizational psychologists, clinical psychologists or psychiatrists, sociologists, behaviorists, managers, Human Resources Managers, and many others. Scientific and academic incubators, in close connection with associations of suitable specialists as well as pro-actively managed (mostly large) organizations, should also be involved in better managing work suffering in the future.

## Author contributions

All authors listed have made a substantial, direct, and intellectual contribution to the work and approved it for publication.

## Conflict of interest

The authors declare that the research was conducted in the absence of any commercial or financial relationships that could be construed as a potential conflict of interest.

## Publisher's note

All claims expressed in this article are solely those of the authors and do not necessarily represent those of their affiliated organizations, or those of the publisher, the editors and the reviewers. Any product that may be evaluated in this article, or claim that may be made by its manufacturer, is not guaranteed or endorsed by the publisher.

